# Quality-of-Life Outcomes in Female Patients With Ileal Conduit or Orthotopic Neobladder Urinary Diversion: 6-Month Results of a Multicenter Prospective Study

**DOI:** 10.3389/fonc.2022.855546

**Published:** 2022-04-12

**Authors:** Salvatore Siracusano, Agustina Zaka, Pierfrancesco Bassi, Paolo Gontero, Ettore Mearini, Ciro Imbimbo, Alchiede Simonato, Fabrizio Dal Moro, Gianluca Giannarini, Claudio Valotto, Francesco Montorsi, Renzo Colombo, Francesco Porpiglia, Riccardo Bartoletti, Marco Vella, Andrea Minervini, Antonio Benito Porcaro, Federico Romantini, Carlo Vicentini, Renato Talamini, Vincenzo Ficarra, Cristina Lonardi

**Affiliations:** ^1^ Department of Life, Health and Environmental Sciences, University of L’Aquila, L’Aquila, Italy; ^2^ Department of Surgery, Dentistry, Pediatrics and Gynaecology, University of Verona, Verona, Italy; ^3^ Department of Medicine and Surgery, Rome Catholic University, Rome, Italy; ^4^ Department of Surgical Science, Turin University, Turin, Italy; ^5^ Department of Medicine and Surgery, Perugia University, Perugia, Italy; ^6^ Department of Urology, Federico II Naples University, Naples, Italy; ^7^ Department of Urology, Palermo University, Palermo, Italy; ^8^ Department of Urology, Padova University, Padova, Italy; ^9^ Department of Urology, Udine Hospital, Udine, Italy; ^10^ Department of Urology, San Raffaele Hospital, Vita e Salute University, Milan, Italy; ^11^ Department of Urology, Orbassano Hospital, Turin University, Turin, Italy; ^12^ Department of Urology, Pisa University, Pisa, Italy; ^13^ Department of Urology, Florence University, Florence, Italy; ^14^ School of Medicine and Surgery, Verona University, Verona, Italy; ^15^ Department of Epidemiology, Aviano Oncology Center, Aviano, Italy; ^16^ Department of Urology, University of Messina, Messina, Italy; ^17^ Department of Human Sciences, Verona University, Verona, Italy

**Keywords:** bladder cancer, female, radical cystectomy, HRQOL, urinary diversion

## Abstract

Radical cystectomy (RC) often affects patients’ life as this surgery is a traumatic and invasive event for the patients, with drawbacks on their daily, social, working, and sex life. Such changes in the quality of life (QoL) of patients are commonly studied through retrospective clinical evaluations and rarely with longitudinal studies. To date, studies focusing on functional outcomes, sexual function, and health-related QoL for female patients are lacking. We evaluated 37 patients using EORTC QLQ-C30 (QLQ-30) and Short-Form 36 (SF-36) questionnaires, before and after surgery, at 3 and 6 months of follow-up. The mean values for the emotional functioning in QLQ-C30 as well as the mental health in SF-36 were significantly higher in the ONB group compared to the IC group at 3 months of follow-up. These differences were not significant at 6 months of follow-up. At 6 months of follow-up, the ONB group showed a higher mean score in the physical and role functioning than the IC group. Although there was a statistically significant age difference at baseline of the two groups, none of the results are correlated with age, as demonstrated by Spearman’s analysis. The ONB seems to represent the most advantageous solution compared to the IC in terms of QOL at the 6-month follow-up.

## Introduction

Bladder cancer (BC) is one of the most frequent cancers in men and women, counting 81,190 new estimated cases in 2018. Radical cystectomy (RC) with lymph node dissection with ileal conduit (IC) or orthotopic neobladder (ONB) urinary diversion is the standard treatment, recommended by the European Association of Urology (EAU) guidelines, for localized muscle-invasive bladder cancer (1). However, RC is also a recommended treatment for high-risk non muscle-invasive bladder cancer (NMIBC) when non-responsive to standard treatments. In this context, it is well known that RC often deeply affects patients’ life, as this surgery is a traumatic and invasive event for the patients, with drawbacks on their daily, social, working, and sex life (2, 3). Such changes and quality of life (QoL) of patients are commonly studied through retrospective clinical evaluations and rarely with longitudinal studies. To date, studies focusing on functional outcomes, sexual function, and health-related QoL for female patients are lacking especially in the short term, although as a whole statistically significant worse overall survival, recurrence-free survival, and cancer-specific survival are reported in comparison to male patients (4). The aim of this study was to evaluate the QoL in female patients in the first 6 months postoperatively with IC or ONB utilizing EORTC QLQ-C30 (QLQ-30) and Short-Form 36 (SF-36) questionnaires in a prospective longitudinal fashion.

## Patients and Methods

This longitudinal study involved 37 consecutive female patients, out of a total of 188 patients of which 151 were males, that had undergone RC and urinary diversion (UD) for urothelial BC in thirteen Italian academic urological centers between September 2019 and July 2020. All patients were older than 18 and were affected by either muscle-invasive BC or by non-responder high-grade non-muscle-invasive BC, according to EAU Guidelines (1). They had all undergone pelvic and iliac lymph node dissection with radical en bloc cystectomy as described by Skinner and Lieskovsky (5) followed by UD by either IC or ONB with Vescica Ileale Padovana (VIP) as previously described by Pagano et al. (6). QoL was measured using the QLQ-C30 and the SF-36 questionnaires before surgery and at 3 and 6 months postoperatively. Baseline characteristics, including demographic profile, body mass index (BMI), Charlson Comorbidity Index (CCI), modified frailty index (m-FI), pathological tumor stage, 90-day complications (7), and neo-adjuvant chemotherapy were collected and compared. To rule out the possible effects of disease-related factors or of the psychological burden of a recent surgical procedure, patients with cancer recurrence or with less than 6 months of follow-up were excluded from the analysis. Patients unable to understand or fill out the questionnaire due to cognitive impairment or insufficient command of the Italian language (four patients) were also excluded. All patients provided written informed consent. The study was approved by the Ethics Committees of Verona and Rovigo (protocol number UQOL1Y) and was conducted in accordance with the principles of research involving human subjects as expressed in the Declaration of Helsinki and the Good Clinical Practice guidelines.

### QoL Questionnaires

All patients were evaluated using the QLQ-C30 and SF-36 questionnaires. The QLQ-C30 is a modular 30-item questionnaire developed and copyrighted by the EORTC as an integrated tool designed to assess the QoL of cancer patients participating in clinical trials. This tool has been translated into 81 languages and used in more than 3,000 studies worldwide. Its cross-cultural validity and reliability have been established. The questionnaire is composed of nine multi-item scales: five functional scales (physical, role, cognitive, emotional and social), three symptom scales (fatigue, pain, nausea/vomiting), a global health and QoL scale, and items assessing the perceived financial burden of cancer and other symptoms frequently reported by cancer patients, such as constipation, diarrhea, dyspnea, loss of appetite, and sleep disturbance (8).

The SF-36 measures different health concepts selected from those used by the Medical Outcomes Study. Through 36 multiple-choice questions, the data are aggregated into 8 scales that investigate physical activity, role and physical health, physical pain, health in general, vitality, social activities, role and emotional state, and mental health (9). There is also a question about the change in health status over the last year. We used the Italian version of SF-36 and of the QLQ-30. In the QLQ-30, the majority of questions were assigned a score from 1 to 4 (1 = not at all, 2 = a little, 3 = quite a bit, 4 = very much). For two questions were assigned a score from 1 to 7 (very poor to excellent). As suggested to the EORTC Manual scoring, we linearly transformed all variables to a 0–100 scale. This manual contains scoring procedures for the QLQ-C30, and it also contains summary information about supplementary modules (EORTC Data Center). The principle for scoring these scales is the same in all cases: (1) estimate the average of the items that contribute to the scale—this is the raw score; and (2) use a linear transformation to standardize the raw score, so that scores range from 0 to 100. For the functional items, the higher score represents a higher level of functioning. For the symptoms/single items, a higher score means a higher level of symptomatology/problems. Data were collected from each of the patients through an individual interview, conducted in the outpatient clinic in the course of a follow-up evaluation visit.

### Statistical Analysis

Mean values with standard deviations ( ± SD) were computed and reported for continuous variables (i.e., age, BMI, Charlson comorbidity, and modified frailty index), and for all items included in the QLQ-C30 and SF-36. The Wilcoxon two-sample test was used to verify differences between continuous variables, whereas the chi-square test was used to compare categorical variables (i.e., gender, levels of education, pathological tumor stage, Clavien–Dindo grade and neoadjuvant chemotherapy). Spearman correlation analysis was used to determine the correlation between age and baseline QoL score. Statistical significance was achieved when the two-sided p-value was 0.05 or less. Statistical analyses were conducted using SAS version 9.3 software (SAS Institute, Inc., Cary, NC, USA).

## Results

Thirty-seven female patients undergoing RC and UD were included in the study. Urinary diversion following RC was IC in 75.6% (28/37) of the population and ONB in 24.3% (9/37). Patients in the ONB group were significantly younger than those in the IC group (mean age 62.8 and 70.2 years, respectively; p = 0.03). Barring that, the two groups did not present statistically significant differences with regard to degree of education, BMI, Charlson comorbidity index, modified frailty index, pathological tumor stage, Clavien–Dindo grade, and neoadjuvant therapy (**Table 1**). In all patients, the QoL was assessed before surgery and 3 and 6 months postoperatively. As far as QoL is concerned and reported in **Table 2**, we found that the mean values for only the emotional functioning in QLQ-C30 as well as the mental health in SF-36 were significantly higher in the ONB group compared to the IC group at 3 months of follow-up. For emotional functioning, the means ( ± SD) were 90.6 ( ± 15.7) and 71.1 ( ± 24.2) respectively (p = 0.02), and for mental health the means were 72.0 ( ± 15.1) and 54.9 ( ± 19.7), respectively (p = 0.02). These differences were not significant at the 6-month follow-up. At the 6-month follow-up, we found that the ONB group compared with the IC group had a higher mean score in the physical and role functioning (QLQ-C30). For physical functioning, the means ( ± SD) were 88.2 ( ± 19.1) and 71.7 ( ± 25.1) respectively (p = 0.05), and for role functioning the means were 90.7 ( ± 12.1) and 62.5 ( ± 33.8), respectively (p = 0.03). A significant lower body pain (SF36) was found in the ONB group compared with the IC group: 84.6 ( ± 21.5) and 61.1 (± 30.9), respectively (p = 0.05). Other items did not yield statistically significant results at 6 months (**Table 2**). Although there was a baseline age difference between the two groups, Spearman correlation analysis showed that none of the significant parameters abovementioned (i.e., physical and role functioning, body pain) were correlated with age (**Table 3**).

**Table 1:** Demographic and pathological characteristics of female patients with RC for localized MIBC according to different urinary diversions: prospective multicenter study in Italy.

Characteristics	ONB	IC	p-value^1^
*Female, N*	*9*	*28*	
*Age (years), mean ( ± SD)*	*62.8 ( ± 6.0)*	*70.2 ( ± 9.0)*	***0.03***
*Education (years), N (%)*			
*5–8*	*2 (22.2)*	*15 (53.6)*	
*9–11*	*2 (22.2)*	*4 (14.3)*	
*≥12*	*5 (55.6)*	*9 (32.1)*	*0.38*
*BMI (kg/m^2^), mean ( ± SD)*	*24.8 ( ± 4.7)*	*23.5 ( ± 5.2)*	*0.40*
*Charlson comorbidity index, mean ( ± SD)*	*1.4 ( ± 1.6)*	*2.6 ( ± 2.1)*	*0.19*
*Modified frailty index, mean ( ± SD)*	*0.8 ( ± 1.0)*	*1.1 ( ± 1.6)*	*0.65*
*Pathological tumor stage, N (%)*			
*Organ confined: ≤pT2, pN0*	*5 (55.6)*	*13 (46.4)*	
*Non-organ confined: pT3–pT4, pN0*	*3 (33.3)*	*7 (25.0)*	
*Lymph node-positive: pN+*	*1 (11.1)*	*8 (28.6)*	*0.56*
*Clavien–Dindo grade, N (%)*			
*I*	*7 (77.8)*	*21 (75.0)*	
*II*	*2 (22.2)*	*4 (14.3)*	
*III–IV*	*–*	*3 (10.7)*	*0.54*
*Neoadjuvant chemotherapy, N (%)*			
*No*	*8 (88.9)*	*24 (85.7)*	
*Yes*	*1 (11.1)*	*4 (14.3)*	*0.81*

Bold value is statistically significant.

**Table 2:** Mean and standard deviation ( ± SD) of EORTC QLQ-C30 and short form 36 scale preoperatively and at each follow-up of female patients with RC for localized MIBC according to different urinary diversion: prospective multicenter study in Italy.

	*Preoperatively (baseline)*			*3 months of follow-up*			*6 months of follow-up*		
	*ONB*	*IC*	*p^1^*	*ONB*	*IC*	*p^1^*	*ONB*	*IC*	*p^1^*
*EORTC QLQ-C30*									
*Global health status*	*63.0 ( ± 19.6)*	*59.6 ( ± 27.2)*	*0.84*	*71.9 ( ± 14.7)*	*58.0 ( ± 24.2)*	*0.14*	*71.3 ( ± 17.2)*	*55.7 ( ± 27.3)*	*0.11*
*Function scale*									
*Physical*	*85.2 ( ± 15.2)*	*81.4 ( ± 18.4)*	*0.65*	*80.8 ( ± 19.3)*	*70.7 ( ± 25.6)*	*0.34*	*88.2 ( ± 19.1)*	*71.7 ( ± 25.1)*	***0.05***
*Role*	*79.6 ( ± 16.2)*	*78.6 ( ± 26.4)*	*0.78*	*83.3 ( ± 23.6)*	*76.3 ( ± 26.7)*	*0.60*	*90.7 ( ± 12.1)*	*62.5 ( ± 33.8)*	***0.03***
*Emotional*	*69.4 ( ± 20.0)*	*65.1 ( ± 24.8)*	*0.70*	*90.6 ( ± 15.7)*	*71.1 ( ± 24.2)*	***0.02***	*83.3 ( ± 17.7)*	*72.0 ( ± 24.3)*	*0.21*
*Cognitive*	*85.2 ( ± 10.0)*	*84.5 ( ± 14.3)*	*0.98*	*93.8 ( ± 12.4)*	*89.3 ( ± 15.9)*	*0.48*	*92.6 ( ± 16.9)*	*83.3 ( ± 22.2)*	*0.14*
*Social*	*81.5 ( ± 25.6)*	*83.9 ( ± 23.3)*	*0.80*	*91.7 ( ± 23.6)*	*78.4 ( ± 25.2)*	*0.14*	*81.5 ( ± 29.4)*	*76.8 ( ± 29.2)*	*0.57*
*Symptom scale*									
*Fatigue*	*33.3 ( ± 11.1)*	*41.3 ( ± 22.3)*	*0.51*	*33.3 ( ± 7.9)*	*42.6 ( ± 22.9)*	*0.62*	*17.3 ( ± 20.9)*	*37.3 ( ± 27.1)*	*0.06*
*Nausea-vomiting*	*16.7 ( ± 0.0)*	*31.5 ( ± 26.9)*	*0.33*	*16.7 ( ± 0.0)*	*35.2 ( ± 29.4)*	*0.55*	*3.7 ( ± 7.4)*	*9.5 ( ± 16.0)*	*0.38*
*Pain*	*40.5 ( ± 21.2)*	*47.1 ( ± 27.2)*	*0.67*	*25.0 ( ± 13.9)*	*41.0 ( ± 24.2)*	*0.15*	*13.0 ( ± 18.2)*	*25.0 ( ± 31.6)*	*0.39*
*Dyspnea*	*33.3 ( ± 0.0)*	*33.3 ( ± 0.0)*	*1.00*	*33.3 ( ± 0.0)*	*33.3 ( ± 0.0)*	*1.00*	*11.1 ( ± 16.7)*	*11.9 ( ± 22.6)*	*0.86*
*Insomnia*	*44.4 ( ± 17.2)*	*60.0 ( ± 29.8)*	*0.30*	*44.4 ( ± 19.3)*	*63.6 ( ± 23.4)*	*0.23*	*18.5 ( ± 33.8)*	*31.0 ( ± 33.9)*	*0.25*
*Appetite loss*	*33.3 ( ± 0.0)*	*55.6 ( ± 29.6*	*0.33*	*33.3 ( ± 0.0)*	*56.7 ( ± 22.5)*	*0.38*	*7.4 ( ± 14.7)*	*14.3 ( ± 24.7)*	*0.84*
*Constipation*	*58.3 ( ± 31.9)*	*55.6 ( ± 33.3)*	*0.86*	*44.4 ( ± 19.3)*	*61.5 ( ± 30.0)*	*0.42*	*18.5 ( ± 24.2)*	*28.6 ( ± 31.1)*	*0.44*
*Diarrhea*	*(±)*	*33.3 ( ± 0.0)*		*33.3 ( ± 0.0)*	*33.3 ( ± 0.0)*	*1.00*	*3.7 ( ± 11.1)*	*11.9 ( ± 24.4)*	*0.37*
*Financial difficulties*	*33.3 ( ± 0.0)*	*52.4 ( ± 26.2)*	*0.38*	*50.0 ( ± 23.6)*	*48.5 ( ± 27.3)*	*0.81*	*14.8 ( ± 24.2)*	*14.3 ( ± 23.0)*	*1.00*
*Short Form 36*									
*Physical functioning*	*68.6 ( ± 35.4)*	*72.7 ( ± 29.8)*	*0.79*	*80.0 ( ± 18.9)*	*63.6 ( ± 25.9)*	*0.21*	*68.9 ( ± 36.3)*	*59.5 ( ± 30.0)*	*0.37*
*Role physical*	*85.0 ( ± 33.5)*	*70.6 ( ± 30.9)*	*0.36*	*100.0 ( ± 0.0)*	*91.1 ( ± 23.2)*	*0.43*	*61.1 ( ± 48.6)*	*43.8 ( ± 46.0)*	*0.36*
*Body pain*	*55.9 ( ± 27.2)*	*69.0 ( ± 29.2)*	*0.25*	*68.0 ( ± 24.0)*	*71.1 ( ± 28.8)*	*0.62*	*84.6 ( ± 21.5)*	*61.1 ( ± 30.9)*	***0.05***
*General health*	*45.3 ( ± 21.0)*	*50.5 ( ± 23.7)*	*0.80*	*51.9 ( ± 8.6)*	*48.0 ( ± 10.2)*	*0.32*	*50.0 ( ± 7.6)*	*46.5 ( ± 10.9)*	*0.33*
*Vitality*	*55.0 ( ± 20.0)*	*45.7 ( ± 22.0)*	*0.28*	*65.0 ( ± 19.6)*	*48.9 ( ± 20.4)*	*0.06*	*55.6 ( ± 19.3)*	*47.5 ( ± 22.8)*	*0.31*
*Social functioning*	*51.4 ( ± 25.3)*	*65.6 ( ± 25.6)*	*0.16*	*65.6 ( ± 20.9)*	*63.8 ( ± 28.9)*	*0.98*	*70.8 ( ± 30.0)*	*58.5 ( ± 29.3)*	*0.25*
*Role emotion*	*83.3 ( ± 33.3)*	*72.9 ( ± 30.4)*	*0.53*	*88.9 ( ± 27.2)*	*74.1 ( ± 29.3)*	*0.24*	*63.0 ( ± 48.4)*	*39.3 ( ± 42.6)*	*0.24*
*Mental health*	*52.0 ( ± 18.6)*	*49.3 ( ± 23.1)*	*0.75*	*72.0 ( ± 15.1)*	*54.9 ( ± 19.7)*	***0.02***	*61.8 ( ± 18.9)*	*56.0 ( ± 23.4)*	*0.46*

Bold values are statistically significant.

**Table 3:** Spearman correlation analysis (r) of correlation between age and preoperative (baseline) 102EORTC QLQ-C30 and short form 36 scale.

QoL scale	ONB		IC	
	r	p	r	p
*EORTC QLQ-C30*				
*Global health status*	*-0.52*	*0.15*	*0.29*	*0.15*
*Function scale*				
*Physical*	*-0.23*	*0.55*	*-0.35*	*0.07*
*Role*	*-0.22*	*0.56*	*-0.07*	*0.73*
*Emotional*	*-0.77*	***0.02***	*0.21*	*0.30*
*Cognitive*	*-0.20*	*0.61*	*0.13*	*0.51*
*Social*	*-0.64*	*0.06*	*0.03*	*0.89*
*Symptom scale*				
*Fatigue*	*0.54*	*0,21*	*-0.02*	*0.92*
*Nausea-vomiting*	*–*	*–*	*-0.75*	***0.02***
*Pain*	*0.51*	*0.24*	*-0.03*	*0.89*
*Dyspnea*	*–*	*–*	*–*	*–*
*Insomnia*	*-0.21*	*0.69*	*0.18*	*0.45*
*Appetite loss*	*–*	***–***	*-0.42*	*0.17*
*Constipation*	*0.21*	*0.79*	*-0.37*	*0.33*
*Diarrhea*	*–*	***–***	*–*	*–*
*Financial difficulties*	*–*	*–*	*-0.18*	*0.70*
*Short Form 36*				
*Physical functioning*	*-0.39*	*0.35*	*-0.17*	*0.40*
*Role physical*	*-0.35*	*0.56*	*0.06*	*0.82*
*Body pain*	*-013*	*0.75*	*0.09*	*0.67*
*General health*	*-0.70*	***0.04***	*-0.05*	*0.80*
*Vitality*	*-0.63*	*0.09*	*0.16*	*0.43*
*Social functioning*	*-0.40*	*0.29*	*-0.12*	*0.55*
*Role emotion*	*-0.26*	*0.74*	*-0.01*	*0.95*
*Mental health*	*-0.29*	*0.48*	*0.15*	*0.46*

Bold values are statistically significant.

## Discussion

According to EAU guidelines, radical cystectomy remains the gold standard treatment for muscle-invasive bladder cancer. The urological literature emphasizes the importance of HRQOL in patients undergoing RC and urinary diversion; however, the information available in this regard is always based on retrospective and non-prospective studies. In this regard, it is known that cross-sectional retrospective studies show various biases that may not reflect the real quality of life of patients. In this context, it is therefore clear that the optimal way is to carry out longitudinal studies possibly randomized with the use of questionnaires validated in the patients’ original language. Currently, the use of prospective studies to evaluate the QoL in patients undergoing RC is not very widespread, as only a few authors have undertaken this methodological approach (3, 10).

In this setting, it is not yet clear whether the HRQOL between patients with continent urinary diversion and incontinent urinary diversion is comparable even if a meta-analysis in this regard would confirm it (11). In our study, we focused our attention on the female population as the data in this regard are particularly lacking due to the lower incidence of BC in female compared to male. We decided to measure the QoL in the first 6 months because scoring data in this postoperative period showed that the main changes on HRQOL following RC would begin at this time of follow-up. In our study, all patients completed the EORTC QLQ-C30 and Short Form 36 questionnaires preoperatively and at 3 and 6 months of follow-up. In particular, at baseline all patients had similar characteristics concerning education, BMI, pathological tumor stage, and Clavien–Dindo grade except for age. In particular, at the 6-month follow-up the global health status does not differ in the two groups of patients although it seems that the ONB group scored significantly higher than in the IC group in the physical and role function scales. Similarly, we observed that the ONB group had a significant lower body pain perception than the IC group. These data were age-independent as shown by Spearman’s analysis, suggesting that at 6 months the QoL begins to be particularly affected in the IC group probably due to the awareness of the care needed for the management of the urinary stoma, possible urinary leak from the bag and urinary odor limiting the ability to partake in social, recreational, and professional activities. In contrast, the ONB group showed a better overall scoring than the IC group most likely related to the presence of a urinary continence allowing to continue an acceptable relationship life. In this way, a previous prospective comparison of HRQOL between patients undergoing RC and IC by Singh (3) showed, unlike what we have found, that global health status is higher in the ONB group than in the IC group and that financial difficulties were significantly greater among patients with IC. Certainly, these two data can be related to the fact that in Singh’s study the prospective analysis involved both sexes overall and that on the other hand the costs and financing of the health system between India and Italy are profoundly different since in Italy any medical expense necessary following the surgical operation would be covered by the national health system. Recently, Clements published the results of a large prospective study showing a detriment of HRQOL at 3 and 6 months with a recovery and stabilization of the same HRQOL at 12 months of follow-up. In this study, the authors found better physical functioning in patients with continent urinary derivation compared to the group with ileal conduit. In our opinion, these results are partially comparable with those reported by us because the Clements study has a follow-up of up to 24 months and because it does not evaluate HRQOL exclusively in women. However, if we analyze the results of Kern, we observe that in the long term and in a perspective way there are no significant differences in HRQOL among non-continent, continent cutaneous, and continent orthotopic urinary diversion groups, which can serve as an important point of consideration during patient counseling (2).

In this context, therefore, we believe that a distinction should be made between HRQOL in the male and female cystectomized patients. In this regard, there are only few studies that try to review recent data on gender differences in oncologic and functional outcomes in women comparing them to male patients. QoL was significantly worse for women than for men (12). A review by Saighian et al. concluded that gender negatively affects oncologic outcome after treatment for bladder cancer, with women being the weaker factor of the equation. In this setting, varying socioeconomic circumstances and biological differences in cancer initiation, as well as response to therapy, seem to be responsible for overall poorer quality of life in bladder cancer female patients, when compared to their male counterpart (13). However, in any case, our study can be considered as the first that exclusively analyzes in a longitudinal way the HRQOL of women undergoing RC with ONB or IC. In this way, it is also important to evaluate the impact of urinary incontinence that may be due to problems related to external appliances. In fact, in cases involving both sexes, leakage with conduit diversions is most commonly due to poor external appliance adherence or suboptimal stoma placement. Among ileal conduit patients, urinary leakage rates during daytime and nighttime have been reported to be as high as 40% (14, 15). Improvements in stomal creation and care, particularly with dedicated enterostomal nurse education, have largely mitigated many of these problems (16), and most patients get to a state of good functional control with minimum urinary leaks after a few months of directed education and gaining hands-on experience changing the urostomy appliance themselves. Although continent urinary diversions are used to preserve normal anatomic urinary function and volitional voiding, urinary incontinence rates and urine leakage are still relatively high. Although most patients regain control during awake hours, nighttime incontinence can affect 40% to 50% of neobladder patients (17, 18). In this context, leakage and lack of control of urinary function can negatively affect HRQOL.

In this context, our study constitutes a contribution to a small body of research addressing an important clinical question on HRQOL for which the ONB seems to represent the most advantageous solution compared to the IC in terms of HRQOL at the 6-month follow-up.

This result can be considered certainly new since to date the retrospective studies conducted in women undergoing RC with ONB or IC have not shown significant differences on HRQOL in the short and mid terms (19); however, about that, it should be borne in mind that QoL results based on interview data may suffer from potential sources of bias that risk inducing responders to report relatively “optimistic” QoLs as patients for example may respond according to what they believe their interviewer wishes to hear.

In conclusion, we can affirm that our study shows some limitations such as the small number of patients enrolled and secondarily that the study was not randomized. These limitations in our case may also be justified by the fact that usually the women who undergo RC are extremely small in number compared to the male population and finally that the randomization in these patients is not feasible because the choice of one or another type of urinary diversion is related to the overall condition of the disease and the general condition of the patient. Further large longitudinal studies will be needed to help the clinicians to understand the real HRQOL of female patients in relation to one or another type of urinary diversion.

## Data Availability Statement

The original contributions presented in the study are included in the article/supplementary material. Further inquiries can be directed to the corresponding author.

## Ethics Statement

The studies involving human participants were reviewed and approved by the Ethics Committees of Verona and Rovigo (protocol number UQOL1Y). The patients/participants provided their written informed consent to participate in this study.

## Author Contributions

SS contributed to the conception and design of the study. AZ organized the database. RT and BP performed the statistical analysis. SS and FR wrote the first draft of the manuscript. PB, PG, EM, CI, AS, FD, GG, CV, FM, RC, FP, RB, MV, AM, CV, VF and CL wrote sections of the manuscript. All authors contributed to manuscript revision and read and approved the submitted version.

## Conflict of Interest

The authors declare that the research was conducted in the absence of any commercial or financial relationships that could be construed as a potential conflict of interest.

## Publisher’s Note

All claims expressed in this article are solely those of the authors and do not necessarily represent those of their affiliated organizations, or those of the publisher, the editors and the reviewers. Any product that may be evaluated in this article, or claim that may be made by its manufacturer, is not guaranteed or endorsed by the publisher.
